# Modified Newcastle Disease virus as an improved vaccine vector against Simian Immunodeficiency virus

**DOI:** 10.1038/s41598-018-27433-x

**Published:** 2018-06-12

**Authors:** Vinoth K. Manoharan, Sunil K. Khattar, Celia C. LaBranche, David C. Montefiori, Siba K. Samal

**Affiliations:** 1 Virginia-Maryland College of Veterinary Medicine, University of Maryland, College Park, Maryland USA; 2 Division of Surgical Sciences, Duke University, Durham, North Carolina USA

## Abstract

SIV infection in macaques is a relevant animal model for HIV pathogenesis and vaccine study in humans. To design a safe and effective vaccine against HIV, we evaluated the suitability of naturally-occurring avirulent Newcastle disease virus (NDV) strains and several modified versions of NDV as vectors for the expression and immunogenicity of SIV envelope protein gp160. All the NDV vectors expressed gp160 protein in infected cells. The gp160 expressed by these vectors formed oligomers and was incorporated into the NDV envelope. All the NDV vectors expressing gp160 were attenuated in chickens. Intranasal immunization of guinea pigs with modified NDV vectors such as rNDV-APMV-2CS/gp160 and rNDV-APMV-8CS/gp160 (NDV strain LaSota containing the cleavage site sequences of F protein of avian paramyxovirus (APMV) serotype 2 and 8, respectively), and rNDV-BC-F-HN/gp160 (NDV strain BC containing LaSota F cleavage site and LaSota F and HN genes) elicited improved SIV-specific humoral and mucosal immune responses compared to other NDV vectors. These modified vectors were also efficient in inducing neutralizing antibody responses to tier 1 A SIVmac251.6 and tier 1B SIVmac251/M766 strains. This study suggests that our novel modified NDV vectors are safe and immunogenic and can be used as vaccine vector to control HIV.

## Introduction

Human immunodeficiency virus-1 (HIV-1) infection can cause acquired immunodeficiency syndrome (AIDS) in humans^1^. According to the Joint United Nations Programme on HIV/AIDS, currently more than 36 million people are infected with HIV globally^2^. Vaccination remains the most efficient approach to control HIV infection in humans. However, a safe and effective vaccine against HIV infection is unavailable. One of the obstacles to develop an effective vaccine against HIV is the lack of a good animal model to test vaccine candidates. Rhesus macaques have been identified as the relevant animal model to evaluate HIV vaccine candidates due to their high degree of genetic relatedness with humans and the similarity between HIV-1 infection in humans and simian immunodeficiency virus (SIV) infection in macaques. Several experimental HIV vaccines have been evaluated in macaques using SIV or simian-human immunodeficiency virus (SHIV) challenge models^3–6^. These include recombinant proteins, peptides, inactivated viruses, DNA and live viral vectored vaccines either alone or in different prime-boost combinations. In parallel, there have been six HIV vaccine efficacy trials in humans that have tested four different vaccine strategies but only RV144 trial showed a modest level of efficacy^7–9^. The data from this trial or from a macaque challenge study^10^ indicated the importance of HIV envelope (Env) protein expressed by recombinant viral vectors and further emphasized the role of antibodies in inducing protective immune responses against HIV.

In the last 30 years, many experimental vaccines have been evaluated for HIV. Among these vaccines, live viral vectored vaccines have shown promising results. To-date, majority of viral vector vaccine studies have been based on viruses such as fowl pox virus, vaccinia virus, adenovirus, vesicular stomatitis virus (VSV) and measles virus^11,12^. However, pre-existing immunity and safety concerns of systemic spread and potential neurovirulence are some of the demerits of these viral vectors^12–14^. Therefore, additional viral vectors that are safe and free from pre-existing immunity in the human population need to be evaluated.

Newcastle disease virus (NDV), an avian virus, belongs to the genus *Avulavirus* in the family *Paramyxoviridae*. NDV is a non-segmented, negative-sense RNA virus, containing six genes, which encode eight proteins. Based on pathogenicity in chickens, NDV strains are classified into three pathotypes: avirulent (lentogenic), moderately virulent (mesogenic) and highly virulent (velogenic)^15^. Natural lentogenic strains LaSota and B1 are used as live vaccines all over the world. NDV has several advantages for use as a vaccine vector for humans^16^. There is no pre-existing immunity to NDV in the human population. It is highly safe in humans. It infects via intranasal route and induces robust local and systemic immune responses^16,17^. Lentogenic NDV strain LaSota has already been evaluated as a vaccine vector in several animal models^17–20^. In addition, mesogenic NDV strain Beaudette C (BC) has been tested as vector in rhesus macaques and it has been shown to replicate to a higher titer, did not cause any disease and induced superior antibody responses compared to the NDV strain LaSota^21^. But mesogenic strain BC is moderately virulent to chickens; therefore, a concern to poultry industry. Currently, both mesogenic and velogenic NDV strains are designated as select agents and hence cannot be used as vaccine vectors. Therefore, we modified the recombinant BC to make it avirulent so that it can be used as a vaccine vector for SIV antigens. This was accomplished either by exchanging complete or part of the genes coding for F and HN proteins with the corresponding genes of the avirulent strain LaSota or by mutating the polybasic residues at F protein cleavage site to either mono or dibasic residues. All the modified BC vectors were found to be avirulent for chickens and met the requirements for exclusion from Select Agent Regulations^15^.

In this study, our goal was to identify the NDV vector that expresses SIV Env gene at the highest level and induces the best immune response. We chose the SIV Env protein for evaluation because it has been shown by several studies that, similar to HIV Env protein, the SIV Env protein is the major antigen against which neutralizing antibodies are produced^22,23^. A series of recombinant (r) NDV vectors expressing gp160 of SIV mac239 strain were used to immunize guinea pigs. The levels of immune response to SIV Env protein expressed by different NDV vectors were evaluated. Our results identified several rNDV/SIV viruses that were highly immunogenic and elicited antibodies that neutralized the SIV mac251.6 strain *in vitro*.

## Methods

### Cells and Viruses

Chicken embryo fibroblast cell line (DF-1), human embryonic kidney cells (HEK 293) and a human epidermoid carcinoma cell line (HEp-2) were obtained from the American Type Culture Collection (ATCC, Manassas, VA). The modified vaccinia virus strain Ankara (MVA) expressing T7 RNA polymerase was kindly provided by Dr. Bernard Moss (NIH, Bethesda, MD). The NDV strains LaSota and the modified rNDVs were propagated in 9-day-old chicken eggs.

### Construction of modified versions of NDV vectors

**Figure 1:**
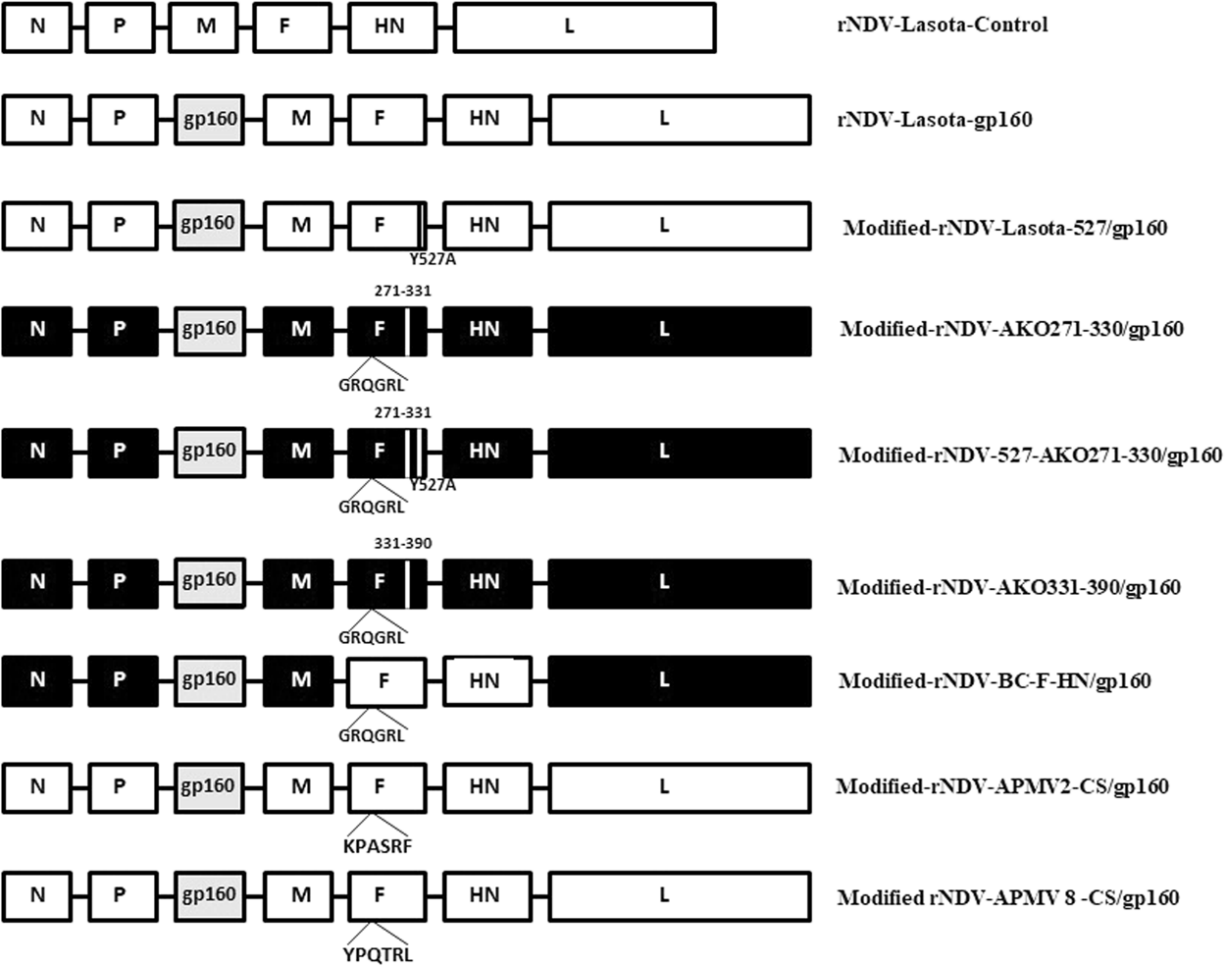
Construction of the modified recombinant NDVs expressing SIV gp160 gene. The SIV gp160 ORF of SIV mac 239 was inserted between P and M genes in the rNDV vectors. Genes derived from rBC or AKO-18 are shown as black or gray rectangles, respectively. F gene segments derived from the NDV strain LaSota strain are shown as white bars. All the viruses contain the avirulent F protein cleavage site sequence of strain LaSota, or APMV-2 or APMV-8.

We used plasmid pNDV carrying the antigenome cDNA of NDV strain LaSota^24^ and modified plasmid pBC of strain Beaudette C (BC) containing some regions of the F gene of NDV strain AKO-18^25^ and plasmid pNDV containing a mutation Y527A in the cytoplasmic tail of F protein^26^, as described previously. In addition, we also made a few modified versions of recombinant pBC containing the F and HN protein of strain LaSota. All the BC recombinants had the avirulent cleavage site as that of strain LaSota (GRQGR↓L) and hence the experiments were carried out in BSL-2. Two modified pNDV- rLaSota vectors containing the avian paramyxovirus serotype-2 (APMV-2) fusion protein cleavage site (KPASR↓F) and avian paramyxovirus serotype-8 (APMV-8) fusion protein cleavage site (YPQTR↓L) were also constructed. They were abbreviated by different names as shown in Fig. 1.

### Construction of rNDV and modified rNDV vectors expressing the SIV envelope glycoprotein (gp160)

The plasmid containing codon optimized envelope gp160 gene of SIV mac239 strain was modified by PCR to contain transcriptional signals of NDV and restriction site for *PmeI*. (Forward: 5′AGCTTTGTTTAAACTTAGAAAAAATACGGGTAGAAGGCCACCATGGGCT130 GCCTGGGCAAC3′, Reverse: 5′GTTTAAACTCACAGCAGGGTCAGTTCC3′). The amplified fragment was cloned into TOPO-TA, sequence confirmed and inserted between the P and M genes of the antigenomic cDNA of strain LaSota (pNDV) and the modified rNDV vectors (Fig. 1). The recombinant viruses were recovered by reverse genetics^17^ and propagated in 9-day-old chicken eggs. To determine the stability of gp160 genes in different rNDV vectors, the recombinant viruses were passaged ten times in chicken eggs and sequence verified.

### Expression of SIV gp160 in cells infected with rNDV and modified rNDVs

To detect expression of SIV gp160, DF-1 cells in 6-well plates were infected with 0.1 MOI of rNDVs containing SIV gp160 and the parental rLaSota virus for 24 h. The cell lysates were analyzed for expression of gp160 by Western blot using a 1:100 dilution of SIV gp120-specific mAb (VM-18S) obtained through the NIH AIDS Reagent Program, Division of AIDS, NIAID, NIH. In order to investigate the incorporation of SIV Env into the NDV virion, allantoic fluid from infected chicken eggs was obtained at 3-day post infection (dpi) and centrifuged at 3,000 rpm for 10 min. The viruses were purified by ultracentrifugation through a 30% sucrose gradient and the pellet containing the viruses was collected and analyzed by Western blot using SIV gp120-specific mAb.

### Analysis of SIV gp160 protein oligomers formation

The formation of oligomers of SIV gp160 expressed by recombinant NDV vectors was determined by protein cross-linking of protein, followed by Western blot analysis^27^. Briefly, monolayers of DF-1 cells were infected with 0.1 MOI of different rNDVs expressing SIV gp160 and rLaSota. After 24 h, cells were harvested and washed twice in PBS. The proteins were cross-linked using 1 mM dithiobis (succinimidyl propionate) (DSP; Pierce), a thiol-cleavable, amine-reactive, and membrane-permeative cross-linker and analyzed under reducing conditions and non-reducing conditions by SDS-PAGE and immunoblotting with gp120-specific mAbs.

### Growth kinetics of rNDVs expressing SIV gp160 in DF-1 cells

The multistep growth kinetics of all the rNDVs were analyzed in DF-1 cells. Cells grown in 6-well plates were infected in duplicate with each rNDVs expressing SIV gp160 at an MOI of 0.001. Supernatant (200 μl) was collected at 8 h intervals until 64 h post infection (hpi). Virus titers in the supernatants were quantified in DF-1 cells by limiting dilution and expressed as 50% tissue culture infective dose per milliliter (TCID_50_/ml)^28^.

### Pathogenicity of rNDVs expressing SIV gp160 in chicks

The pathogenicity of the different rNDVs expressing SIV gp160 was determined by the intracerebral pathogenicity index (ICPI) test in 1-day-old SPF chicks^29^. In the ICPI test, 0.05 ml of a 1: 10 dilutions of fresh egg-grown virus was injected into group of ten 1-day-old SPF chicks by the intracerebral route. At each 12 h observation, birds were scored 0 if normal, 1 if sick, and 2 if dead. The ICPI is the mean score per bird over 8-day observation period.

### Immunization of guinea pigs

Groups of 5-week-old female guinea pigs (3 animals/group) were immunized with either the parental vector (rLaSota/gp160) or modified rNDVs expressing SIV gp160 (100 μl each of 1 × 10^5^ TCID_50_) via intranasal route after xylazine and ketamine anesthesia. The control group was inoculated with rLaSota without any SIV gene. All the animals received one booster dose of immunization after 3-weeks. Blood was collected at different time intervals (days 0, 14, 21, 28, 35, 42, 49, 56 and 63). Vaginal wash samples were also collected and processed as described previously^27^.

### Serum and mucosal antibody responses

The SIV gp160 specific antibody responses were analyzed using enzyme-linked immunosorbent assays (ELISAs). ELISA plates (96 well), were coated overnight with a purified recombinant gp130 protein of SIV mac239 (received from NIH AIDS Reagent Program, Division of AIDS, NIAID, NIH)^30^ at a concentration of 1 μg/ml in 100 μl volume/well, in sodium carbonate-bicarbonate buffer (pH 9.8). The plates were then blocked with 3% skim milk and 2% sucrose^27^. Serum samples or vaginal wash samples were diluted in dilution buffer (Zoetis Inc, Kalamazoo, MI) and added to the plates. The plates were incubated for 1 h with a 1:1000 dilution of an isotype-specific secondary antibody, horseradish peroxidase (HRP)-conjugated goat anti-guinea pig IgG (Seracare, Millford, MA), goat anti-guinea pig IgG1, goat anti-guinea pig IgG2a (Novus Biologicals, Littleton, CO), or sheep anti-guinea pig IgA (Immunology Consultants Laboratory, Newberg, OR). After washing, the plates were developed with ABTS (2,2′-azinobis [3-ethylbenzothiazoline-6-sulfonic acid]-diammonium salt) peroxidase substrate solution (Zoetis Inc, Kalamazoo, MI). The plates were analyzed at 405 nm using an ELISA plate reader.

### Virus neutralization assay

Neutralizing antibody levels were measured using a pseudovirus based assay in TZM-bl cells^31^. Envelope pseudotyped viruses of tier 1 A SIVmac251.6, tier 1B SIVmac251/M766 strains and tier 2 SIVmac239CS.23 were made by transfecting the appropriate plasmids in 293 T/17 cells^31^. Two-fold serial dilutions of heat inactivated serum samples were made in culture media and added to 96-well plates (10 μl/well). Then 200 TCID_50_ of pseudovirus in 40 μl volume was added to each well. After incubation for 1 h at 37 °C, TZM-bl cells were added to each well (1.5 × 10^4^/well in 150 μl volume of culture media containing 10% heat-inactivated FBS and DEAE-dextran). Assay controls included TZM-bl cells alone (cell control) and TZM-bl cells with virus and no antibody (virus control). After 48 h incubation at 37 °C, the medium was removed from each well and replaced with 50 μl of RPMI 1640 medium (Invitrogen) and 50 μl of BriteLite plus Reagent (Perkin Elmer). The cells were lysed for 2 min and 100 μl of cell lysate was transferred to a 96-well black microtiter plate, and luminescence was measured. The 50% inhibitory dose (ID_50_) titer was measured.

### Ethics Statement

All the animal experiments were conducted in our Bio Safety Level-2 + facility according to the protocols approved by Institutional Animal Care and Use Committee of the University of Maryland.

## Results

### Generation of rNDVs expressing SIV gp160

The transcription cassette containing the ORF of SIV mac239 gp160 gene was inserted between the P and M genes as an additional gene-cassette into the antigenomic cDNA of lentogenic NDV strain LaSota and other modified NDV vectors (Fig. 1). All the rNDVs were recovered by standard reverse genetic technique^24^. The designation of each rNDV carrying SIV mac239 gp160 gene is described in Fig. 1. The recovered viruses were passaged 10 times in 9-day-old chicken eggs and the presence of the foreign gene was confirmed by sequence analysis.

### Expression of SIV gp160 protein by rLaSota and modified rNDVs

**Figure 2:**
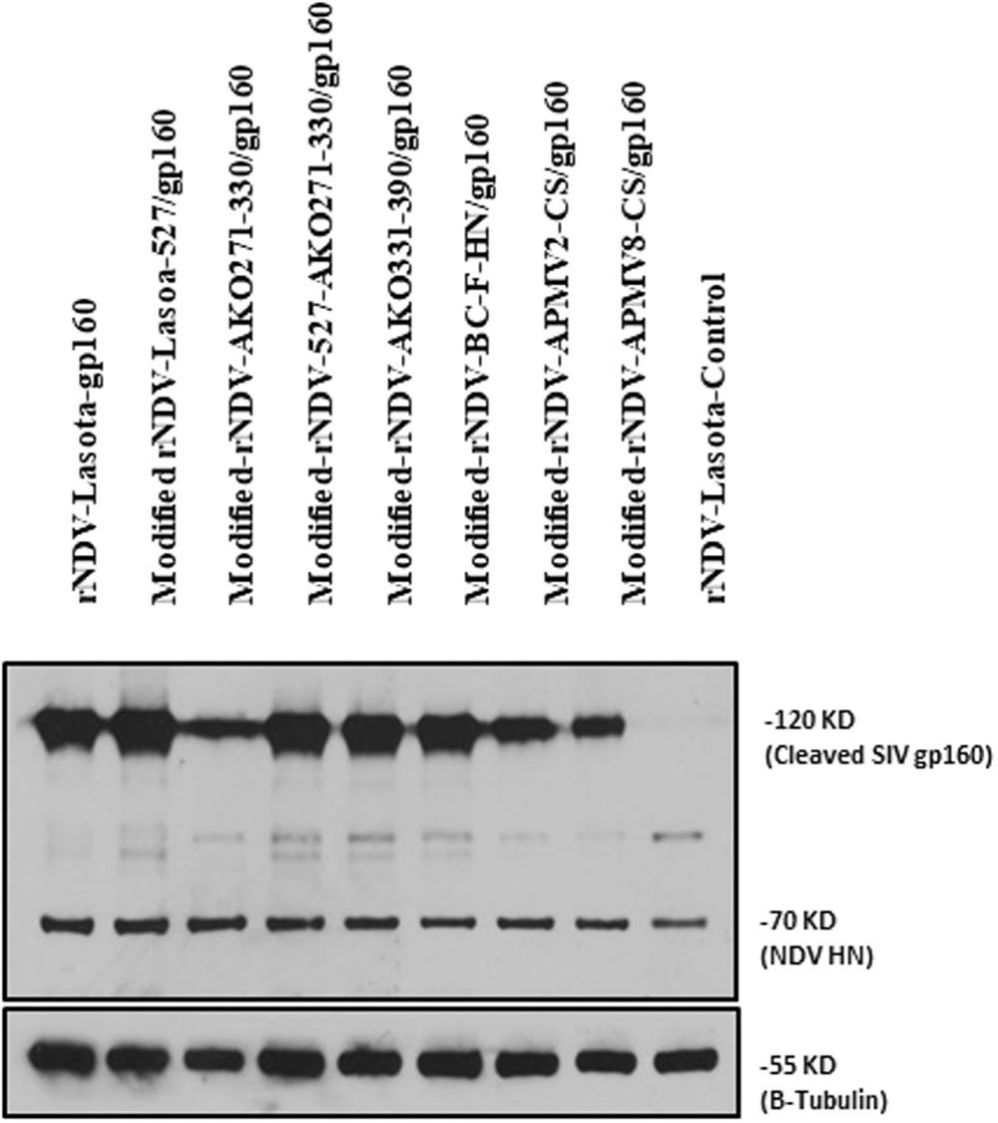
Expression of SIV gp160 *in vitro* by modified rNDV vectors. Cell lysates were collected from DF-1 cells infected with each virus at an MOI of 1. Western blot analysis was performed using gp120-, HN- and β-tubulin- specific mAbs to detect SIV gp160, NDV HN and cellular β-tubulin proteins.

**Figure 3:**
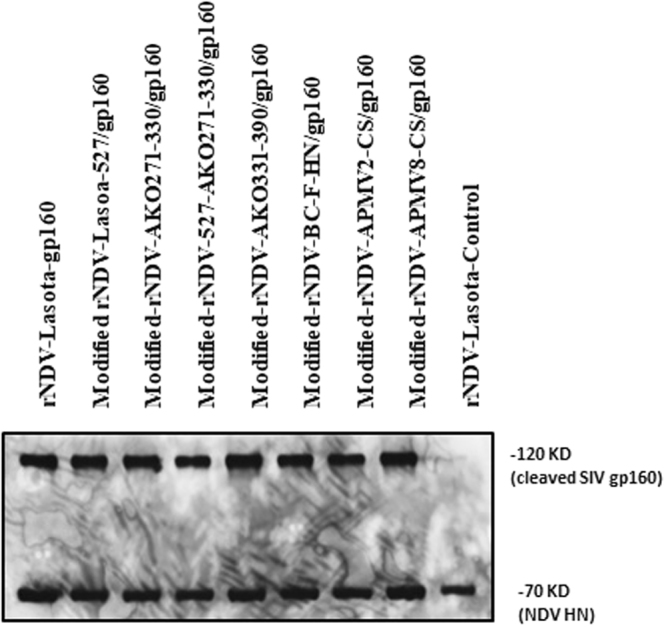
Incorporation of the SIV gp160 into rNDV particles. The allantoic fluid containing each virus was harvested from eggs and passed through 30% sucrose cushion. The sedimented virus was suspended in PBS and used for Western blot analysis. Monoclonal antibodies specific to SIV gp160 and NDV HN were used to detect these proteins. Molecular weight of the detected virus proteins (in kDa) are shown.

To detect expression of the SIV gp160, DF-1 cell monolayers were infected with rLaSota and modified rNDVs containing SIV gp160 gene. DF-1 cell lysates were analyzed by Western blot using gp120 specific mAb. All the rNDVs expressed the cleaved gp160 Env protein of 120 kDa in size (Fig. 2). The densitometric analysis (data not shown) revealed that the level of gp160 expressed by different rNDVs varied slightly, with modified-rNDV-LaSota-527/gp160 showing highest level of expression followed by modified-rNDV-AKO-331-390/gp160. To determine the incorporation of SIV gp160 into NDV particle, the allantoic fluid harvested from eggs infected with rNDVs was ultracentrifuged through a 30% sucrose cushion and the purified virus was analyzed by Western blot using gp120-specific mAb (Fig. 3). Western blot analysis showed that SIV gp160 was incorporated into NDV particle. The insertion of SIV gp160 gene into the rNDV genome did not alter the expression of HN protein present on NDV envelope, as shown by similar levels of HN protein expression by all rNDVs.

### Oligomer formation of SIV gp160 expressed by rNDV

**Figure 4:**
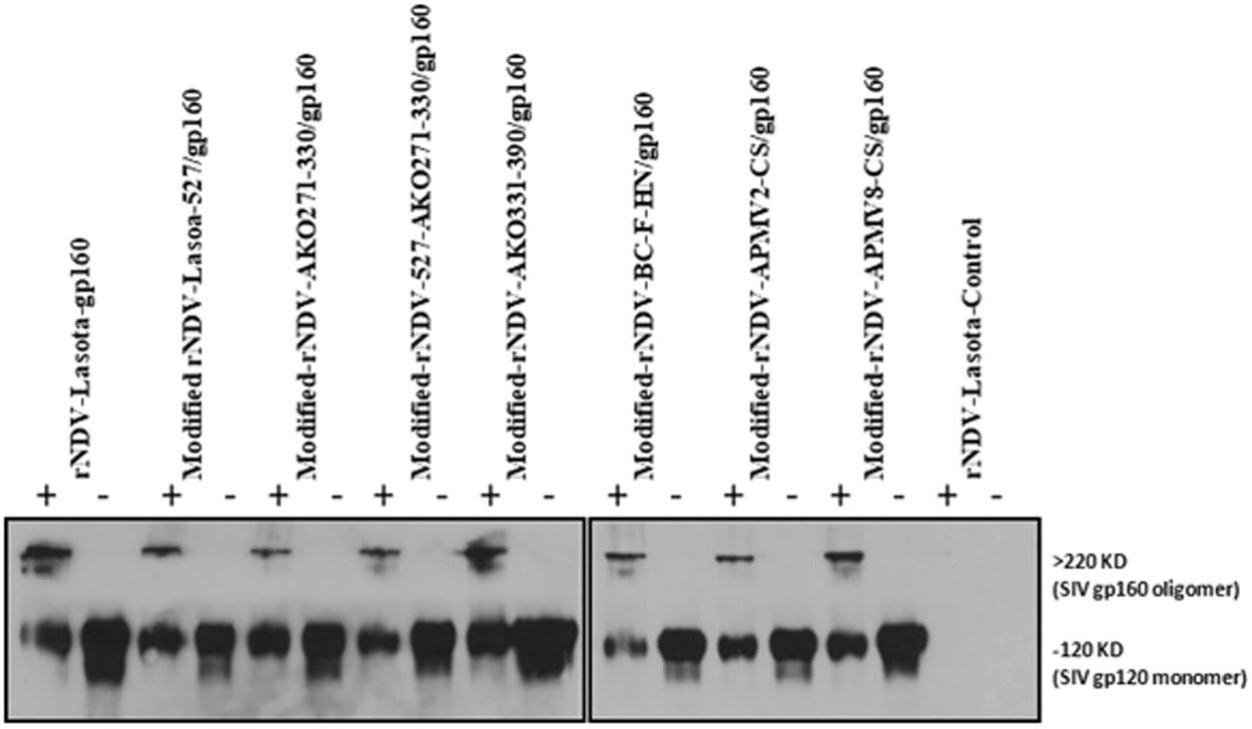
Oligomer formation of of SIV-1 gp160. Lysates of DF-1 cells infected with modified rNDVs expressing SIV gp160 were cross-linked with DSP and analyzed by SDS-PAGE under reducing (−) or non-reducing (+) conditions. Monomeric gp120 and oligomeric gp160 (shown on right margin) expressed by different rNDV vectors were detected after immunblotting with SIV gp120-specific monoclonal antibodies. Two gels were run so as to include all the 18 lysates of infected DF1 cells.

The envelope protein of SIV assembles to form noncovalent associated oligomers. The oligomeric complex plays an important role in virus entry and is the major target of the neutralizing antibodies^23^. Hence, we determined the oligomerization of the gp160 expressed by different modified NDV vectors in infected DF-1 cells. Cell lysates from NDV-infected DF-1 cells were cross-linked with DSP followed by SDS-PAGE analysis in reducing and non-reducing conditions followed by Western blotting with gp120-specific antibodies. The presence of oligomers of higher molecular weight (>220 kDa) under nonreducing conditions and monomers (120 kDa) under reducing conditions is shown in Fig. 4. These results suggest that rLaSota and modified rNDVs support the expression of SIV gp160 as one predominant oligomer of size greater than 220 kDa.

### Biological characterization of rNDV expressing gp160

**Figure 5:**
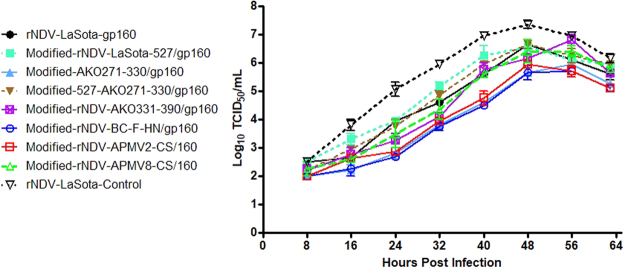
*In Vitro* growth kinetics of rLaSota and modified rNDV vectors expressing SIV gp160 in DF-1 cells. DF-1 Cells were infected with their respective viruses at 0.001 MOI, in the presence of 10% fresh chicken egg allantoic fluid and 2% FBS. Cell culture supernatant samples were harvested every 8 h intervals until 64 h post infection and titrated by TCID_50_ assay in DF-1 cells. The graph shows the geometric mean ± standard error of the mean (SEM) of TCID_50_ values.

The growth kinetics of the rLaSota and modified rNDVs expressing SIVgp160 were compared in DF-1 cells (Fig. 5). Compared to parental rLaSota without any insert, the replication of rLaSota and modified rNDVs containing SIVgp160 gene was reduced. Although there were differences in the growth kinetics of modified rNDVs, all the viruses containing the SIVgp160 gene insert reached to similar titers at 64 h post infection. The pathogenicities of modified NDVs expressing SIVgp160 and the parental rLaSota virus was evaluated by the ICPI test in 1-day-old chicks. The ICPI values of all rNDVs and modified rNDVs were 0.00 suggesting that wild-type rNDV and modified rNDVs expressing SIVgp160 are avirulent and the insertion of foreign gene had no major effect on virus growth and pathogenicity.

### Evaluation of humoral immune responses in guinea pigs

**Figure 6:**
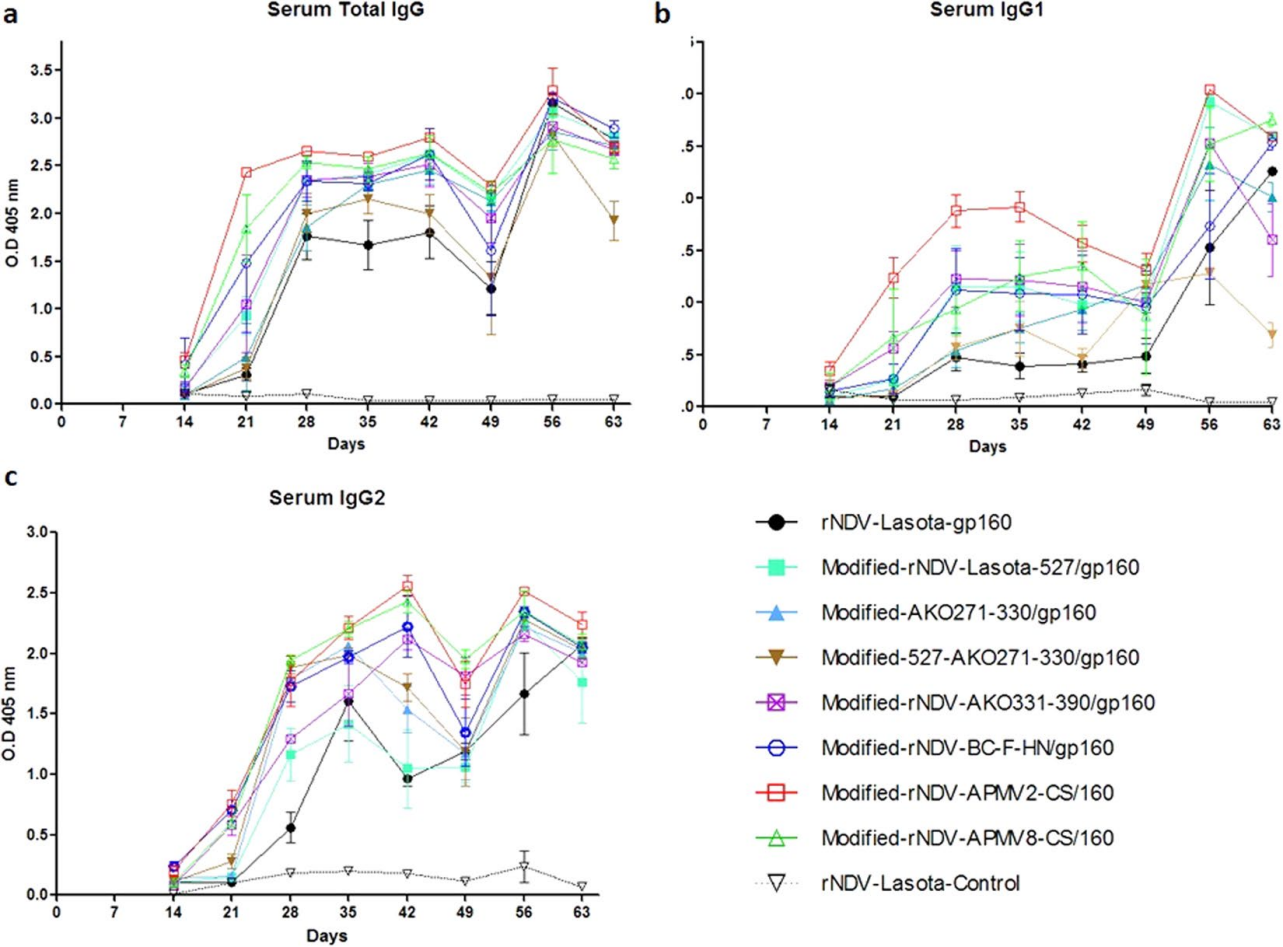
Serum antibody responses against SIV gp160 in guinea pigs. Serum samples (1:1000), collected from immunized guinea pigs at the indicated time point, were analyzed for anti-SIV gp120 antibodies using ELISA. Arrows indicate times of rNDV immunizations on days 0 and 21. The geometric mean ± standard error of the mean (SEM) for 3 animals/group are shown in each graph.

Nine groups of female Hartley guinea pigs (n = 3) were immunized on days 0 and 21 with rLaSota and modified rNDVs expressing SIV gp160 via intra nasal route. The animals in all the groups remained healthy without any clinical signs. The induction of SIV Env-specific total IgG, IgG1 and IgG2a in serum was measured in all groups on days 14, 21, 28, 35, 42, 49, 56, and 63 by ELISA (Fig. 6). Barring rNDV-LaSota control group, Env-specific antibody responses were detected on day 14 in all the groups. The total IgG, IgG1 and IgG2a antibody responses increased between days 28 and 42 in all groups. Total IgG response peaked on day 56 before decreasing on day 63. The IgG1 responses decreased on day 49 in all groups except in rNDV-LaSota-gp160 and in modified-527-AKO271-330/gp160 groups and peaked on day 56 before decreasing on day 63. Similarly, IgG2a responses decreased on day 49 and increased on day 56 except the wild type rNDV group. The gp160-specific IgG response observed with the modified-rNDV-APMV2-CS/gp160, modified-rNDV-BC-F-HN/gp160 and modified rNDV-APMV8-CS/gp160 groups were significantly higher than rNDV-LaSota gp160 group. The highest IgG titer was observed in modified-rNDV-APMV2-CS/gp160 group. The antibody titers in other groups were slightly higher than the rLaSota group. These results indicated that SIV Env-specific IgG responses could be enhanced by using Modified-rNDV-APMV-2-CS/160, Modified-rNDV-APMV-8-CS/160 and Modified-rNDV-BC-F-HN/160 vectors.

### Evaluation of mucosal immune responses in guinea pigs

**Figure 7:**
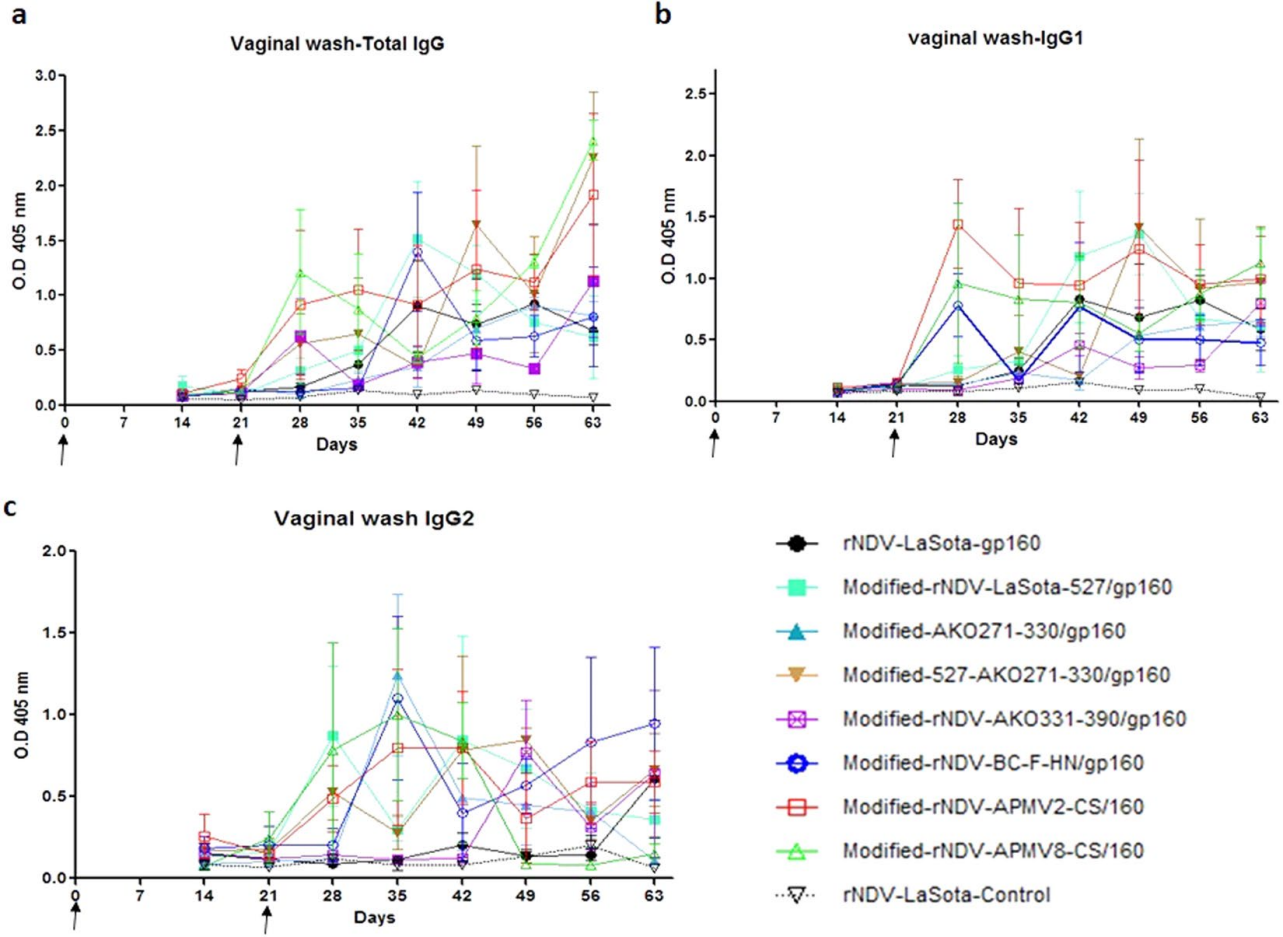
Mucosal antibody responses against SIV gp160 in guinea pigs. Vaginal wash samples collected from guinea pigs at indicated time points were diluted in PBS (1:10) and analyzed by ELISA for SIV gp120-specific antibodies. Arrows indicate times of rNDV immunizations on days 0 and 21. The geometric mean ± standard error of the mean (SEM) for 3 animals/group are shown in each graph.

Vaginal wash samples were evaluated for antibody responses by ELISA using SIV gp130-coated plates (Fig. 7). Very low total IgG, IgG1 and IgG2a antibody titers were detected on day 21, but the antibody responses increased significantly in all groups after the boost except in the rNDV-LaSota control group. The titer of total IgG, IgG1 and IgG2a were highest between days 28 and 63. Similar to IgG responses in serum, the response was highest in the modified-rNDV-APMV2-CS/gp160 group.

### Evaluation of neutralizing antibody responses in guinea pigs

**Figure 8:**
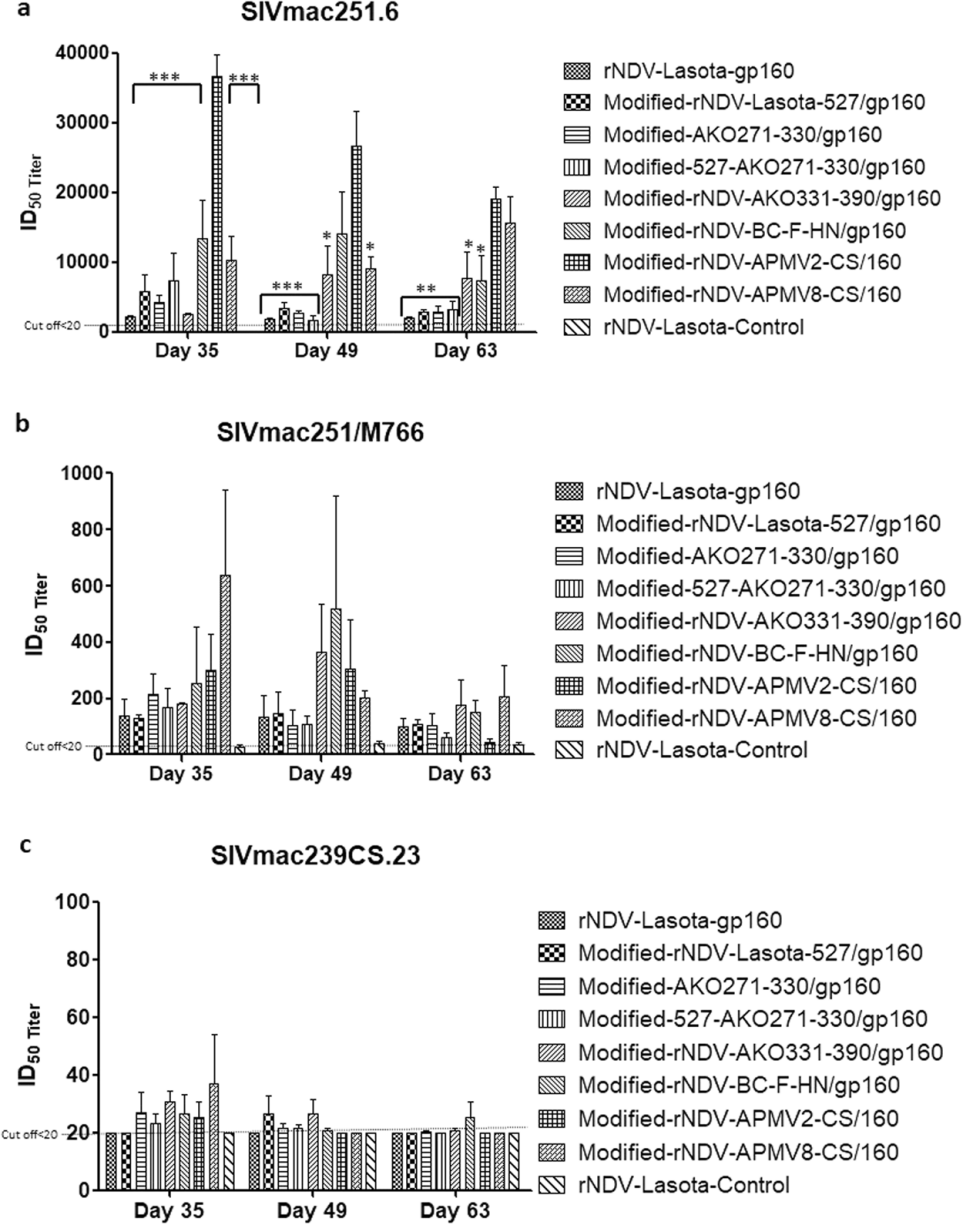
Neutralizing antibody activity represented as ID_50_ (50%-inhibitory-dilution) against heterologous tier 1 A SIV mac251.6, tier 1B SIV mac 251/M766 and homologous tier 2 strain SIV mac239cs.23. Serum samples from immunized guinea pigs were obtained on days 35, 49 and 63 and tested for neutralization of pseudoviruses by the TZM-bl assay. Serum obtained from negative control group (rNDV-LaSota-Control) was used as as an indicator of background signal. The dashed lines denote background titers of <20. The Statistical significance between different groups were compared to serum samples from modified-rNDV-APMV2-CS/gp160, which showed highest neutralizing antibody titers, with Tukey’s multiple comparison test. The geometric mean ± standard error of the mean (SEM) for the 3 animals/group are shown in each graph. ^*^P value < 0.05; ^**^P value < 0.005; ^***^P value < 0.001 are indicated on each graph.

Serum samples were evaluated for neutralization titer against tier 1 A SIVmac251.6, tier 1B SIVmac251/M766 and tier 2 SIVmac239CS.23 HIV strains on days 35, 49, and 63 by TZM.bl assay (Fig. 8). Neutralizing antibody (nAb) titer (expressed as ID_50_) was detected against SIVmac251.6 in all groups with the highest ID_50_ values in modified-rNDV-APMV2-CS/160 group on days 35, 49 and 63 followed by modified-rNDV-BC-F-HN/gp160 group on days 35 and 49 and modified rNDV-APMV8-CS/160 group on day 63. The nAb response in the modified-rNDV-APMV2-CS/160 group peaked on day 35. The nAb activity detected against SIVmac251/M766 was higher in the rNDV-APMV8-CS/160 group followed by modified-rNDV-APMV2-CS/160 and modified-rNDV-BC-F-HN/gp160. A very low response to tier 2 SIVmac239CS.23 was detected, consistent with the highly neutralization-resistant phenotype of this virus^32^. The nAb response to SIVmac251.6 was significantly higher than the response to SIVmac251/M766 in all the guinea pig groups.

## Discussion

We have previously shown that lentogenic NDV strain LaSota expressing HIV-1 Env protein gp160 induces strong mucosal and serum antibody responses in guinea pigs^27^. In another study we used the NDV strain LaSota to compare the biochemical immunological characteristics of Env proteins gp160, gp140 and gp120^33^. We found that similar to gp160, gp140 and gp120 formed oligomers which were recognized by conformation-sensitive monoclonal antibodies. Our results showed that NDV strain LaSota expressing gp140 induced higher antibody responses compared to the other recombinants in guinea pigs. We have also shown that immunization of guinea pigs with NDV strain LaSota co-expressing Env and Gag proteins elicited potent immune responses to HIV^17^. All the above studies showed that lentogenic strain LaSota is a promising vaccine vector for HIV. However, these results need to be confirmed in a relevant animal model in which challenge studies can be performed. Therefore, in this study we have expressed SIV Env protein using several NDV vectors and compared their immune responses in guinea pigs. We have identified a modified NDV vector that induces higher antibody responses in guinea pigs. This vector needs to be evaluated in macaques by SIV challenge.

In this study, we have used rNDV as a vaccine vector for SIV Env protein because of several reasons^16^. NDV is antigenically distinct from human pathogens. Most humans are not exposed to NDV and hence there is no pre-existing immunity. NDV grows in Vero cell line which is certified for vaccine production. Vaccines derived from NDV vectors can be given nasally and can induce effective humoral and mucosal immune responses. NDV has an intrinsic immune-modulatory property for the induction of efficient immune responses. Compared to lentogenic strains, mesogenic strains of NDV have been found to mount an effective immune response in non-human primates^21^. However, mesogenic strains are Select Agents and must be handled in BSL-3 facility which makes development and testing of mesogenic NDV vectors challenging. Therefore, in this study, the polybasic F cleavage site of mesogenic strain BC was modified to the dibasic F cleavage site of lentogenic strain LaSota. The change in F cleavage site reduced the virulence of mesogenic strain BC suggesting that it has the potential to be used as a vaccine vector. Changing the F protein cleavage site of lentogenic strain LaSota to those of APMV-2, 7 and 8 also eliminated the need for exogenous protease supplementation, while still containing avirulent F protein cleavage site. These modifications were done to improve the replication ability of strain LaSota and hence enhance its usage as a vaccine vector. The modified viruses were avirulent by ICPI test and induced higher levels of immune responses against SIV Env protein in guinea pigs.

Several reports have shown that an effective neutralizing antibody response conferred protection against HIV in different animal models. However, non-neutralizing antibodies also played a significant role in decrease of incidence of HIV-1 infection in phase III RV144 vaccination trial^7,8^. In this study, we have developed several modified rNDV vectors and have used these vectors to express SIVgp160 mac239 envelope protein. The expression of SIV gp160 in DF-1 cells was higher in modified-rNDV-LaSota-527/gp160 followed by the modified-NDV-rBC vectors. Studies have shown that foreign envelope proteins expressed by rNDV gets incorporated into NDV envelopes^17^. In this study. We found that SIV gp160 also got inserted into the envelope of NDV particle. An important advantage of the SIV envelope protein incorporation into NDV particles is that they can effectively mount potent immune responses.

SIVmac239CS.23 and its closely related derivatives SIVmac251.6 and SIV mac251/M788 vary widely in their ability to neutralization with the same antisera^34^. rNDV vectors expressing SIVmac239 gp160 induces neutralizing antibodies to strain SIVmac251.6 and SIVmac251/M788 whereas the same antibodies rarely neutralize infectivity of SIVmac239CS.23. This might be due to a subset of antibodies that bound with an affinity too low to neutralize the SIVmac239CS.23. The current notion is that, more easily neutralized viruses have a more open conformation Env glycoprotein, while the Env glycoprotein of more neutralization-resistant viruses exist primarily in a more closed conformation^35^. The variations in the neutralization titer might be also due to the antigenic heterogeneity among different SIV strains. SIV infects diverse hosts and it is possible that the virus evolves having its own neutralizing determinants^32^. SIVmac239CS.23 was remarkably resistant to neutralization even with homologous anti-sera induced by our rNDV vectors. It could be due distinct molecular determinants of neutralization of this virus strain compared to SIVmac251.6 and SIV mac251/M788 virus strains^36^. The serum antibody levels were increasing from day 28–56 as evident by ELISA results, but the nAb titers were decreasing over time. The reduction in nAb titer over time might be due to lack of persistent infection, which is essential for the clonal selection of high-affinity antibodies. We measured levels of antibodies present in vaginal secretions, because mucosal antibodies play an important role in prevention of sexual transmission of HIV and SIV. Immunization with rNDV vectors expressing SIVgp160 elicited gp120-specific IgG in the vaginal wash samples of all of the animals. In addition, the NDV vectored SIV vaccines used in this study elicited nAbs against SIV strains different from vaccinated strain.

The major drawback of most viral vectors for vaccination is the interference of their replication after homologous virus boosting^37,38^. Usage of pox virus, vaccinia virus and adenovirus as viral vectors are limited by this drawback. It can be overcome by use of either heterologous viral vector or use of homologous viral vector with modifications. Therefore, we modified NDV vector in this study to test immunogenicity against SIV Env protein. Some of these modified vectors have shown improved immunogenicity and protective efficacy in prime-boost vaccination in our previous studies^25,39,40^. Although we have not tested these modified NDV vectors for prime-boost vaccination in this study, we expect these vectors to produce better immune responses against SIV or HIV proteins in future prime-boost vaccination studies.

In summary, we compared different modified rNDV vectors expressing SIVgp160 by prime and boost strategy for induction of serological and mucosal antibody responses to tier 1 A SIVmac251.6, tier 1B SIVmac251/M766 and tier 2 SIVmac239CS.23 strains. These rNDV vectors induced higher levels of nAbs in guinea pigs, without causing any clinical signs. Modified-rNDV-APMV2-CS/gp160 stimulated nAb response to SIVmac251/M766 at a higher magnitude than other modified vectors in guinea pigs. Previous studies have shown that SIVmac239CS is highly neutralization resistant which explains the low neutralizing titers by all the rNDV vectors^32^. We have identified three rNDV vectors that induced higher levels of humoral immune responses against SIV. The potential of these vectors needs to be tested in other animal models such as macaques.
